# Intradiscal Delivery of Notoginsenoside R1‐Loaded PLGA Nanoparticles Attenuates Intervertebral Disc Degeneration in Rats

**DOI:** 10.1111/jcmm.71378

**Published:** 2026-09-23

**Authors:** Baichuan Zheng, Haotian Zhong, Zhengwei Wang, Junyu Qin, Ziying Xie, Rong Tang, Chengshang Hu, Xiuming Zhang, Jianwen Li, Songbo Li

**Affiliations:** ^1^ The Tenth Affiliated Hospital Southern Medical University (Dongguan People's Hospital) Dongguan Guangdong China; ^2^ Guangdong Medical University Zhanjiang Guangdong China; ^3^ The Affiliated Dongguan Songshan Lake Central Hospital Guangdong Medical University Dongguan Guangdong China; ^4^ Shenzhen School of Clinical Medicine Southern Medical University Shenzhen China; ^5^ Dongguan Key Laboratory of Basic Clinical and Digital Research on Common Orthopedic Diseases Dongguan Guangdong China

**Keywords:** extracellular matrix homeostasis, intervertebral disc degeneration, notoginsenoside R1, oxidative stress, PLGA nanoparticles

## Abstract

Intervertebral disc degeneration (IVDD) contributes to persistent low back pain, but existing treatments focus on symptom relief rather than addressing the degeneration. Notoginsenoside R1 (NR1), a bioactive saponin derived from Panax notoginseng, has exhibited protective effects in experimental models of disc injury. However, its clinical application is limited by poor bioavailability and rapid clearance. Here, NR1 was encapsulated into poly(lactic‐co‐glycolic acid) nanoparticles (NR1@PLGA) to improve local drug retention. NR1@PLGA showed uniform particle characteristics, prolonged NR1 release, and time‐dependent uptake by nucleus pulposus cells (NPCs). Under H_2_O_2_‐induced oxidative stress, NR1@PLGA reduced SA‐β‐gal‐positive cells, preserved aggrecan and collagen II (Col‐2) expression, and improved cell viability compared with free NR1 at the same concentration. In a rat caudal puncture model, intradiscal administration of NR1@PLGA preserved T2‐weighted MRI signals, improved histological outcomes, increased aggrecan expression, and decreased MMP‐13 levels. No obvious abnormalities were observed in major organs or serum biochemical indicators related to liver and kidney function during the 4‐week observation period. These results demonstrate that NR1@PLGA is a potential treatment for IVDD, with PLGA encapsulation enhancing the efficacy of NR1.

## 1 Introduction

Low back pain (LBP) affected approximately 619 million people worldwide in 2020, and this number is projected to increase to 843 million by 2050 [1, 2]. The substantial contribution of LBP to years lived with disability (YLDs) also places a considerable socioeconomic burden on healthcare systems and society [3, 4]. Among the different causes of LBP, intervertebral disc degeneration (IVDD) is acknowledged as a significant pathological change driving disease progression [5]. IVDD progresses due to the gradual malfunction of nucleus pulposus cells, leading to disrupted extracellular matrix balance and disc structural damage [6, 7]. This process is further aggravated by excessive oxidative stress, persistent inflammation and abnormal ECM metabolism [8, 9]. Current therapeutic strategies for IVDD are largely limited to conservative pharmacological management, which involves the use of NSAIDs, analgesics and muscle relaxants, or surgical interventions, such as discectomy, intervertebral fusion and total disc replacement [10, 11]. Despite their clinical benefits, these approaches do not effectively interrupt disease progression or restore disc homeostasis [10, 12]. Therefore, new therapeutic approaches targeting IVDD progression are still needed.

NR1, a panaxatriol saponin from Panax notoginseng (Burk.) F.H. Chen, is known for its antioxidant, anti‐inflammatory and cytoprotective effects [13]. Numerous studies have demonstrated the obvious protective influence of NR1 in many diseases, such as inflammatory bowel disease, myocardial infarction and osteoarthritis. NR1 has also been reported to exert tissue‐protective effects in multiple organ systems, indicating its potential for ameliorating organ injury [13, 14, 15, 16, 17, 18]. More recently, NR1 has also been reported to attenuate nucleus pulposus cell injury, suppress inflammatory responses and pyroptosis through suppression of the NF‐κB/NLRP3 pathway, thereby delaying the development of IVDD [19]. In addition, NR1 has been reported to exert antioxidant effects by reducing oxidative stress and ROS accumulation in various diseases [20, 21, 22]. Despite these promising findings, the therapeutic application of free NR1 remains limited by its poor physicochemical stability, rapid clearance and low bioavailability, which substantially restrict its local therapeutic efficacy [23, 24, 25].

**FIGURE 1:**
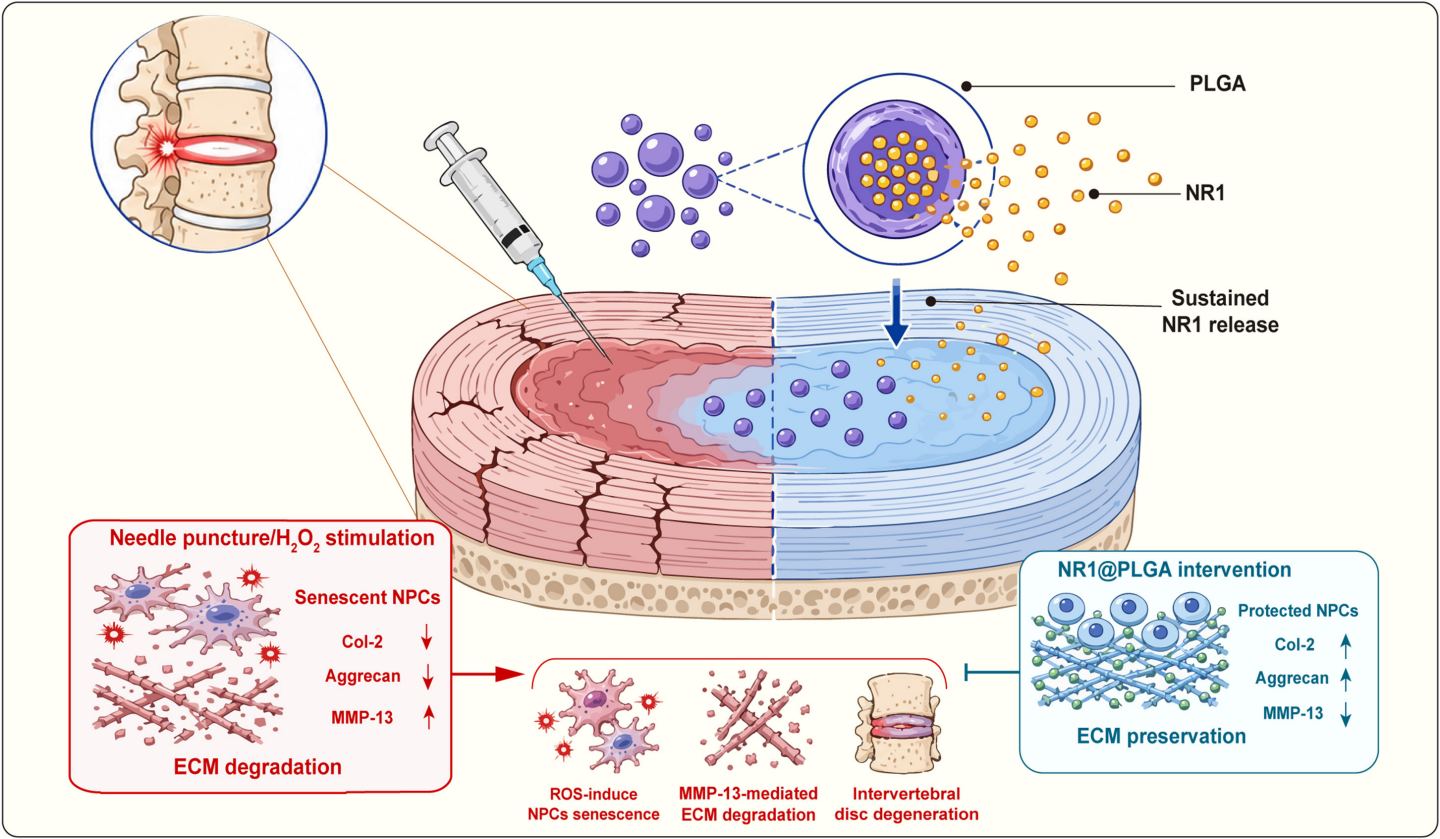
Schematic illustration of the design and therapeutic strategy of NR1@PLGA for IVDD.

The use of nanoparticles in drug delivery has provided new opportunities to improve the stability, retention, and release profiles of bioactive compounds [10, 26]. Owing to its excellent biocompatibility, biodegradability, and safety, PLGA has been widely studied as a biodegradable carrier [27, 28]. Studies have demonstrated that PLGA nanoparticles facilitate the encapsulation of small‐molecule drugs and regulate their release profiles, contributing to improved drug stability and local therapeutic effects [29, 30]. In this study, NR1‐loaded PLGA nanoparticles (NR1@PLGA) were prepared, and their physicochemical characteristics and protective effects against IVDD were evaluated in H_2_O_2_‐treated NPCs and a rat caudal puncture model (Figure 1).

## 2 Materials and Methods

### 2.1 Materials

NR1 was obtained from Shanghai Macklin Biochemical Technology Co. Ltd. (Shanghai, China). PLGA was obtained from Xi'an Ruixi Biological Technology Co. Ltd. (Xi'an, China). The SA‐β‐gal staining kit and CCK‐8 were obtained from Beyotime Biotechnology Co. Ltd. (Shanghai, China). Antibodies against matrix metalloproteinase‐13 (MMP‐13), Col‐2 and aggrecan were obtained from Proteintech Group Inc. (Wuhan, China). Four percent paraformaldehyde and EDTA decalcification solution (pH 7.2) were purchased from Biosharp Life Sciences (Beijing, China). Serum alanine aminotransferase (Cat. No. S03030), aspartate aminotransferase (Cat. No. S03040), creatinine (Cat. No. S03076), and urea (Cat. No. S03036) assay kits were purchased from Rayto Life and Analytical Sciences Co. Ltd. (Shenzhen, China).

### 2.2 Preparation of PLGA Nanoparticles

The preparation of NR1@PLGA nanoparticles involved a modified emulsification‐solvent evaporation approach as previously described. Briefly, an organic solution containing PLGA (60 mg) and NR1 (5.5 mg) in 1.2 mL of dichloromethane was prepared and slowly introduced into 20 mL of 5% (w/v) PVA solution. The mixture was emulsified by intermittent probe sonication in an ice bath for 60 s (30 s at 100 W followed by 30 s at 200 W). The emulsion was maintained under magnetic stirring at 300 rpm in an open container for 3–4 h to facilitate the removal of dichloromethane. Then, the emulsion was centrifuged at 4000 rpm for 5 min to remove large aggregates, followed by centrifugation of the supernatant at 12,000 rpm for 10 min to collect the nanoparticles. The remaining supernatant was subjected to repeated centrifugation under the same conditions to maximize nanoparticle recovery. The collected nanoparticle pellets were washed twice with deionized water, resuspended in 10 mL of purified water. After adding a cryoprotectant, the NR1@PLGA nanoparticle suspension was lyophilized and stored at −20°C until use. PLGA nanoparticles without NR1 were prepared using the same method.

### 2.3 Characterization of NR1@PLGA Nanoparticles

PLGA and NR1@PLGA nanoparticles suspensions were diluted 20‐fold with purified water before analysis. Nanoparticle size distribution and polydispersity index (PDI) were evaluated by dynamic light scattering (DLS), and zeta potential measurements were performed by phase analysis light scattering using a NanoBrook Omni analyser (Brookhaven Instruments, USA). The structure of NR1@PLGA nanoparticles was examined using transmission electron microscopy (TEM). For sample preparation, carbon‐coated copper grids were used to hold diluted nanoparticle suspensions for 30 s. Filter paper was used to remove the excess liquid, and then it was dried at room temperature.

For NR1 quantification, a methanolic stock solution of NR1 (1 mg/mL) was serially diluted to prepare standard solutions with concentrations of 30, 35, 45, 50, 80, and 100 μg/mL. Then, standard solutions were analysed by high‐performance liquid chromatography (HPLC) with UV detection at 203 nm. Plotting the peak area against NR1 concentration resulted in a calibration curve (*R* ^2^ = 0.9986). The amount of NR1 encapsulated in PLGA nanoparticles was then quantified by HPLC. The freeze‐dried NR1@PLGA nanoparticles were dissolved in dichloromethane, and then the solvent was evaporated using nitrogen. The resulting residue was dissolved in methanol and passed through a 0.22 μm membrane before HPLC measurement. The formulas used to calculate encapsulation efficiency (EE) and drug loading (DL) are as follows: EE (%) = (amount of NR1 encapsulated/initial amount of NR1) × 100%, and DL (%) = (amount of NR1 encapsulated/total weight of freeze‐dried nanoparticles) × 100%.

### 2.4 In Vitro NR1 Release Studies

The release pattern of NR1 from NR1@PLGA nanoparticles in vitro was assessed through a dialysis method, using a free NR1 solution as the control. Briefly, NR1@PLGA nanoparticle suspension or free NR1 solution containing an equivalent amount of NR1 was transferred into dialysis bags and submerged in PBS under continuous shaking. Samples were taken from the release medium at specific intervals and replaced right away with the same amount of fresh PBS to keep sink conditions. Released NR1 was quantified by HPLC, and the cumulative release profile was calculated based on the initial NR1 content in the dialysis bags.

### 2.5 Animal Experiments

In this study, 8‐week‐old male SD rats were used. All methods received approval from the Experimental Animal Ethics Committee at the Tenth Affiliated Hospital of Southern Medical University (Dongguan People's Hospital), under Approval No. IACUC‐AWEC‐202605010. Rats were randomly assigned to five groups (five rats per group): Control, Model, PLGA, NR1, and NR1@PLGA. Rats in the Control group received neither needle puncture nor local treatment. Rats in the remaining four groups underwent puncture of the Co8/9 and Co10/11 caudal intervertebral discs. Under intraperitoneal anaesthesia with 2.5% tribromoethanol, the tail was disinfected with povidone iodine, and the target intervertebral disc levels were identified by x‐ray imaging. A sterile 23‐gauge needle was inserted into each target disc to a depth of 4.5 mm, rotated 360°, maintained in place for 1 min, and then withdrawn. After puncture, 3 μL of PBS, blank PLGA nanoparticle, free NR1, or NR1@PLGA nanoparticle suspension was injected into each target disc in the Model, PLGA, NR1, and NR1@PLGA groups, respectively.

### 2.6 MRI Examination

Four weeks after needle puncture and local treatment, the caudal intervertebral discs were examined using a 3.0‐T MAGNETOM Skyra MRI system (Siemens Healthineers, Germany). T2‐weighted images were acquired to assess the hydration and structural integrity. The degree of disc degeneration at the Co8/9 and Co10/11 levels was assessed according to the Pfirrmann grading system by two independent blinded observers.

### 2.7 Biochemical Serum Analysis

Following MRI examination, blood was collected from the abdominal aorta under anaesthesia, and serum was separated for biochemical analysis. The levels of creatinine (CREA), alanine aminotransferase (ALT), aspartate aminotransferase (AST), and blood urea nitrogen (BUN) were determined in accordance with the manufacturer's guidelines.

### 2.8 Histological Analysis

After MRI examination, the rats were anaesthetised. The harvested intervertebral disc specimens and major organ samples were preserved in 4% paraformaldehyde overnight. Disc tissues were decalcified in an EDTA‐based decalcification solution for 2 months, with the solution replaced every 3 days. After decalcification, the tissues underwent routine histological processing, including graded ethanol dehydration, xylene clearing, paraffin embedding, and sectioning at a thickness of 3 μm. Major organ samples were processed by routine paraffin embedding without decalcification. Histological evaluation of intervertebral disc sections was performed using H&E, Safranin O/Fast Green, and toluidine blue staining. H&E staining was used on paraffin sections of the heart, liver, spleen, and kidneys. The stained sections were scanned and digitized with a digital pathology slide scanner. Histological changes in the intervertebral discs were evaluated according to the Rutges histological scoring system, and quantitative image analysis was performed using ImageJ2 software.

### 2.9 Immunohistochemistry

The harvested tissue samples were embedded in paraffin and microtome‐sliced. To remove paraffin, the sections were treated with xylene and subsequently rehydrated using ethanol solutions with decreasing concentrations. The process of antigen retrieval involved digesting with pepsin at 37°C for 30 min. Sections were then exposed to primary antibodies for MMP‐13 and aggrecan at 4°C overnight and incubated with appropriate secondary antibodies at room temperature. DAB was used for signal development, and nuclei were counterstained with haematoxylin. The sections were subsequently dehydrated, cleared, mounted, and scanned using a digital pathology slide scanner.

### 2.10 Cell Culture

Primary NPCs were obtained from SD rat nucleus pulposus tissues. Under sterile conditions, the tissues were dissected, cut into small pieces, and transferred into sterile centrifuge tubes. Collagenase II solution (0.2%, prepared in complete DMEM/F12 medium and filtered through a 0.22 μm membrane) was added at 7 mL per tube according to the tissue volume. The samples were incubated at 37°C with gentle shaking for 30 min, with additional digestion performed when tissue fragments remained visible. The digested cell suspension was collected and maintained in complete DMEM/F12 medium supplemented with 10% FBS and 1% penicillin–streptomycin.

### 2.11 Cell Viability Assay

NPCs were placed into 96 well plates at a density of 5 × 10^3^ cells per well. After the indicated treatments, the CCK‐8 reagent was mixed with serum‐free DMEM/F12 medium at a 1:9 (v/v) ratio to prepare the working solution. The solution was then added to the cells and incubated for 60 min at 37°C. Absorbance was recorded at 450 nm using a microplate reader.

### 2.12 Immunofluorescence Staining for Col‐2 and Aggrecan

After treatment, NPCs underwent fixation and permeabilization before incubation with 5% bovine serum albumin for 1 h. Primary antibodies targeting Col‐2 or aggrecan were applied at 4°C overnight. On the following day, cells were washed with PBS and incubated with fluorophore‐labelled secondary antibodies for 1 h. The fluorescence images were taken with an inverted fluorescence microscope (Zeiss LSM 900, USA).

### 2.13 Senescence‐Associated Beta‐Galactosidase Staining

After treatment, NPCs were washed with PBS and then fixed for 15 min at room temperature. The cells were incubated in an SA‐beta‐gal staining solution at 37°C. Bright field images were captured with an inverted microscope. Cells exhibiting distinct blue cytoplasmic staining were considered SA‐β‐gal‐positive. Five non‐overlapping fields were randomly selected from each sample, and the percentages of positive cells were calculated.

### 2.14 Statistical Analysis

Results are presented as mean ± SD, and statistical analyses were executed with GraphPad Prism 9.0. For datasets meeting the assumptions of parametric tests, differences among multiple groups were analysed by one‐way ANOVA. Otherwise, Dunn's multiple‐comparison test was conducted after the Kruskal‐Wallis test. Pfirrmann grades and histological scores were analysed using non‐parametric tests. *p* < 0.05 was considered statistically significant.

## 3 Results

### 3.1 Characterization of NR1@PLGA Nanoparticles

**FIGURE 2:**
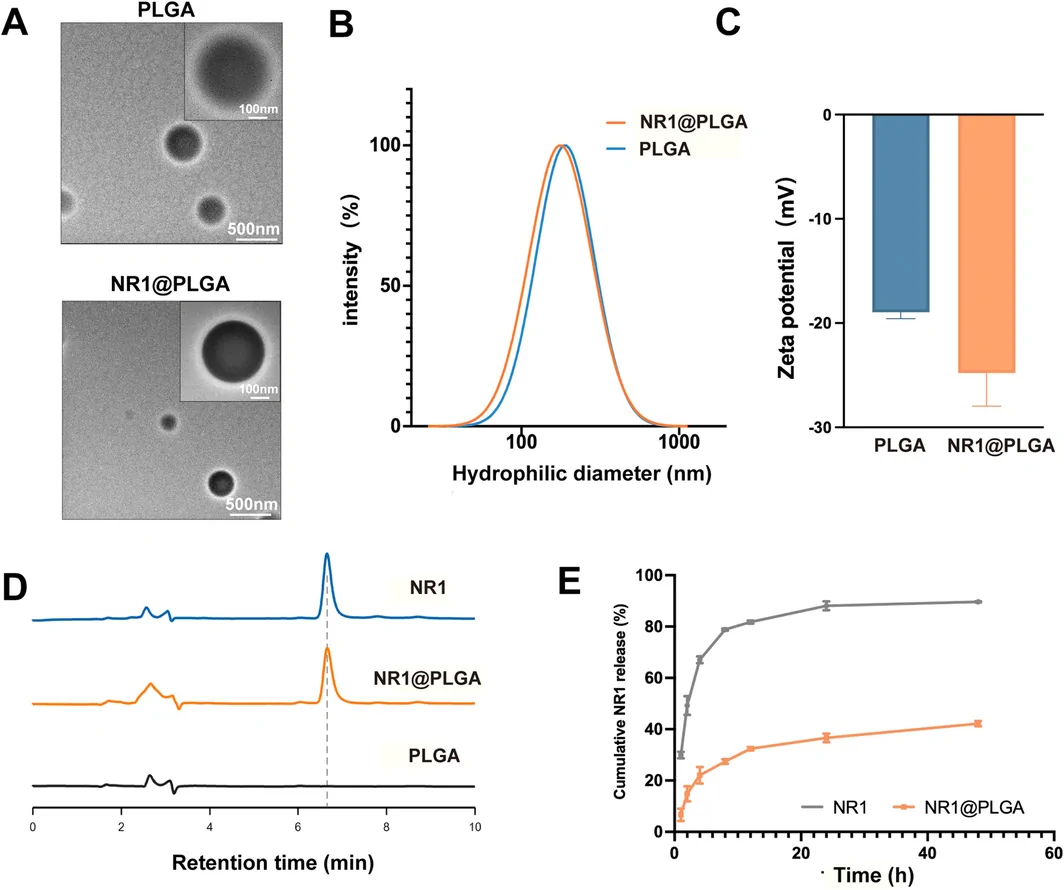
Characterization of NR1@PLGA nanoparticles. (A) TEM image of PLGA nanoparticles before and after loading NR1. (B) Hydrodynamic size distribution of PLGA nanoparticles before and after loading NR1. (C) Zeta potentials of PLGA nanoparticles before and after loading NR1. (D) HPLC chromatograms of NR1, blank PLGA and NR1@PLGA. (E) In vitro cumulative release profiles of free NR1 and NR1@PLGA in PBS (pH 7.4).

Blank PLGA and NR1@PLGA nanoparticles were prepared, followed by characterization of their physicochemical properties. As shown in Figure 2A, both nanoparticles had a consistent spherical morphology with smooth surfaces and no significant clumping. DLS analysis revealed a relatively narrow particle size distribution for both formulations. The mean hydrodynamic diameters were 197.82 ± 17.90 nm for PLGA nanoparticles and 198.20 ± 17.93 nm for NR1@PLGA nanoparticles, indicating that encapsulating NR1 had little influence on particle size (Figure 2B). Negative zeta potentials were measured for both nanoparticle formulations, with values of −18.96 ± 0.60 mV for blank PLGA and −24.79 ± 3.18 mV for NR1@PLGA (Figure 2C). HPLC chromatograms identified a clear NR1 peak in NR1@PLGA, while no corresponding peak was observed in blank PLGA, supporting successful loading of NR1 into the PLGA nanoparticles (Figure 2D). An HPLC standard curve was generated for NR1. It exhibited good linearity over the tested concentration range (*R* ^2^ = 0.9986) (Figure S1). Based on the standard curve, the drug loading and encapsulation efficiency of NR1@PLGA were determined to be 5.60% and 61.04%, respectively. The physicochemical characterization results of blank PLGA and NR1@PLGA, including particle size, PDI, zeta potential, drug‐loading content, and encapsulation efficiency, are summarized in Table S1. The in vitro release behaviour of NR1 was further investigated. NR1 was released more slowly from NR1@PLGA than from the free NR1 formulation. After 48 h, the cumulative release of free NR1 reached 89.64% ± 0.23%, whereas only 42.17% ± 1.04% of NR1 was released from NR1@PLGA, indicating that PLGA encapsulation effectively slowed NR1 release and enabled sustained drug release (Figure 2E).

### 3.2 Cellular Uptake of NR1@PLGA Nanoparticles by Nucleus Pulposus Cells

**FIGURE 3:**
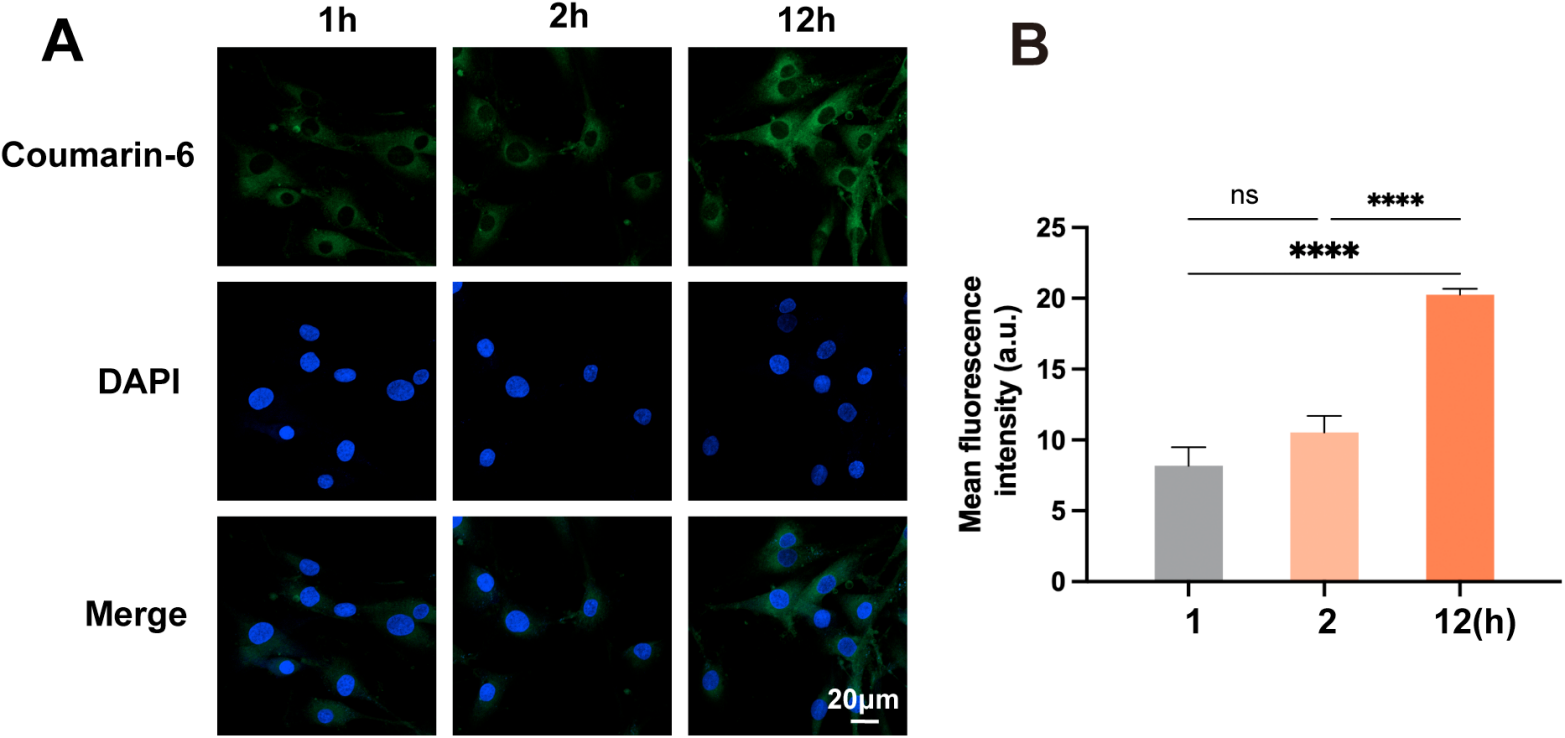
Cellular uptake of Coumarin‐6‐loaded PLGA nanoparticles by NPCs. (A) Representative CLSM images of NPCs incubated with Coumarin‐6‐loaded PLGA nanoparticles for 1, 2 and 12 h. Green: Coumarin‐6, Blue: DAPI. (B) Quantitative analysis of Coumarin‐6 fluorescence levels over different incubation periods. Data are presented as mean ± SD (*n* = 3). ****p < 0.0001, ns, not significant.

Cellular uptake of NR1@PLGA nanoparticles was assessed using Coumarin‐6 (C6)‐labelled nanoparticles. NPCs were incubated with the labelled nanoparticles for 1, 2, and 12 h. As shown in Figure 3, intracellular green fluorescence was observed in NPCs after 1 h of incubation and gradually increased at 2 and 12 h, indicating progressive intracellular accumulation of the nanoparticles. Analysis of quantitative fluorescence intensity showed that nanoparticle uptake increased over time, reaching its peak intracellular fluorescence intensity at 12 h. These results indicated efficient internalization of NR1@PLGA nanoparticles by NPCs, highlighting their suitability for intracellular drug delivery.

### 3.3 NR1@PLGA Alleviates Oxidative Stress‐Induced NPC Senescence and Preserves Extracellular Matrix Homeostasis

**FIGURE 4:**
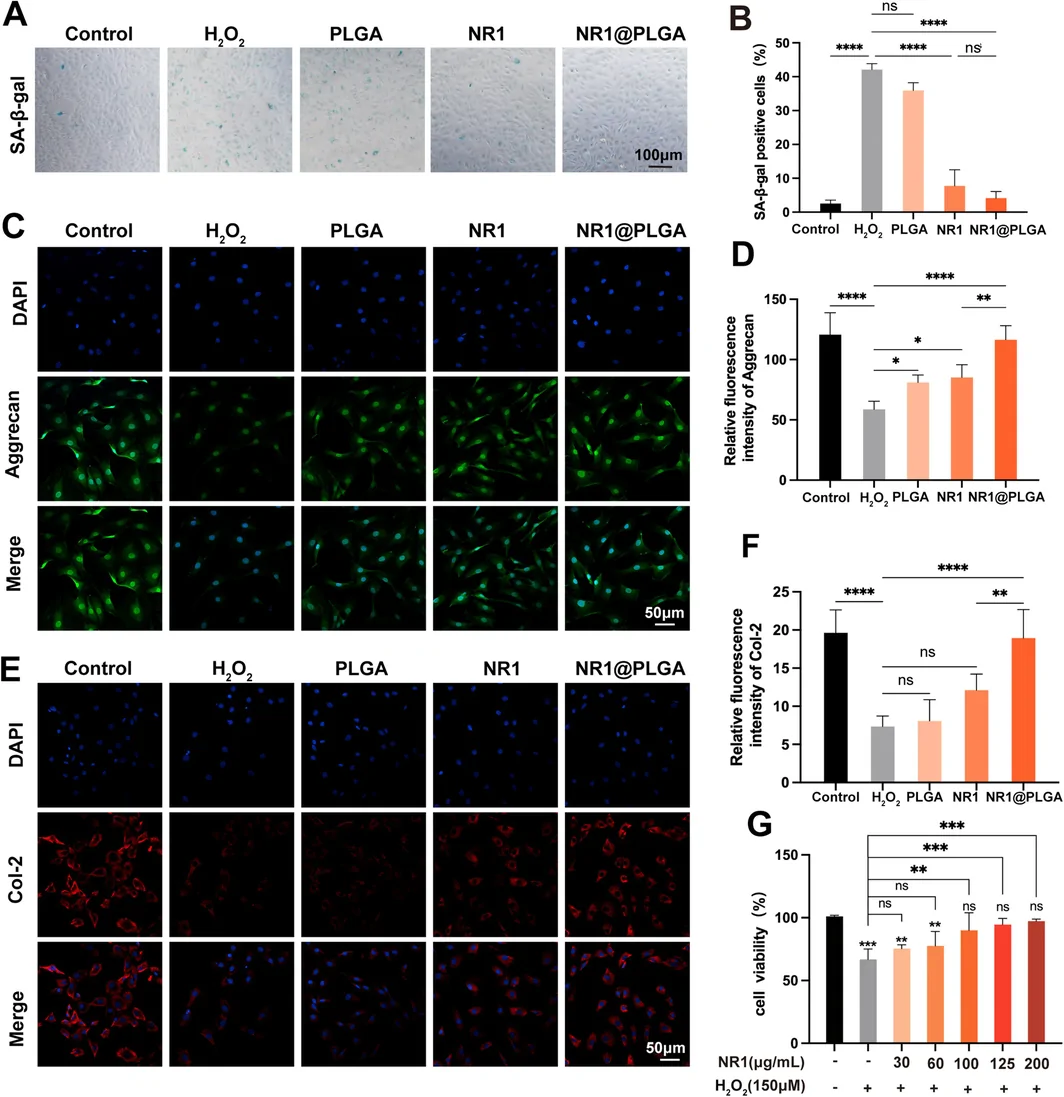
NR1@PLGA nanoparticles protect NPCs against H_2_O_2_‐induced senescence and ECM loss. (A) Representative images of SA‐β‐gal staining in NPCs following the indicated treatments. (B) Quantification analysis of SA‐β‐gal‐positive cells. (C) Representative immunofluorescence images of aggrecan (green) in NPCs following the indicated treatments. (D) Quantitative analysis of aggrecan fluorescence intensities. (E) Representative immunofluorescence images of Col‐2 (red). (F) Quantitative analysis of Col‐2 fluorescence intensities. Except for the control group, all groups were exposed to H_2_O_2_, and the PLGA, NR1, and NR1@PLGA groups received the corresponding treatments under H_2_O_2_ stimulation. (G) The CCK‐8 assay in NPCs following the indicated treatments. Data are presented as mean ± SD (*n* = 3). *p < 0.05, **p < 0.01, ***p < 0.001, ****p < 0.0001; ns, not significant.

The cytotoxicity of NR1@PLGA was first evaluated by treating NPCs with different concentrations of NR1@PLGA using the CCK‐8 assay. No significant changes in cell viability were observed at the tested concentrations (Figure S3). The effects of oxidative stress on cell damage were studied by treating NPCs with rising doses of H_2_O_2_, followed by evaluating cell viability with the CCK‐8 assay. As shown in Figure S2, H_2_O_2_ reduced NPC viability in a concentration‐dependent manner. Based on these results, the optimal H_2_O_2_ concentration was selected for subsequent experiments. NPCs were then challenged with H_2_O_2_ and treated with blank PLGA nanoparticles, free NR1, or NR1@PLGA. To investigate the effect of NR1@PLGA on oxidative stress, we used DCFH‐DA (a fluorescent probe for ROS) to detect changes in intracellular ROS levels by fluorescence microscopy. The green fluorescence intensity reflects the intracellular ROS level. As shown in Figure S4, H_2_O_2_ treatment increased the green fluorescence intensity in NPCs, whereas treatment with free NR1 or NR1@PLGA significantly reduced ROS levels under H_2_O_2_ treatment. Cellular senescence was evaluated by SA‐β‐gal staining. As shown in Figure 4A,B, H_2_O_2_ exposure markedly increased the percentage of SA‐β‐gal‐positive cells, whereas free NR1 treatment partially reduced this effect. Notably, NR1@PLGA resulted in fewer senescent cells than free NR1, indicating greater protection against H_2_O_2_‐induced NPC senescence (Figure 4A,B). To evaluate the impact of NR1@PLGA on ECM balance, immunofluorescence staining was used to examine the expression of aggrecan and Col‐2, which are key matrix components of the nucleus pulposus [9, 31]. As shown in Figure 4C–F H_2_O_2_ stimulation decreased the expression of aggrecan and Col‐2. Compared with free NR1, NR1@PLGA more effectively recovered the levels of both proteins. The protective effects of NR1 and NR1@PLGA under H_2_O_2_ stimulation were further assessed using the CCK‐8 assay. As shown in Figure 4G, NR1 treatment restored cell viability in a concentration‐dependent manner under H_2_O_2_ exposure. NR1@PLGA treatment also increased cell viability in a concentration‐dependent manner under H_2_O_2_ exposure (Figure S5). These findings demonstrate that PLGA‐mediated delivery enhances the protective effects of NR1 against oxidative stress‐induced NPC senescence while preserving extracellular matrix homeostasis.

### 3.4 NR1@PLGA Attenuates Puncture‐Induced IVDD in Rats

**FIGURE 5:**
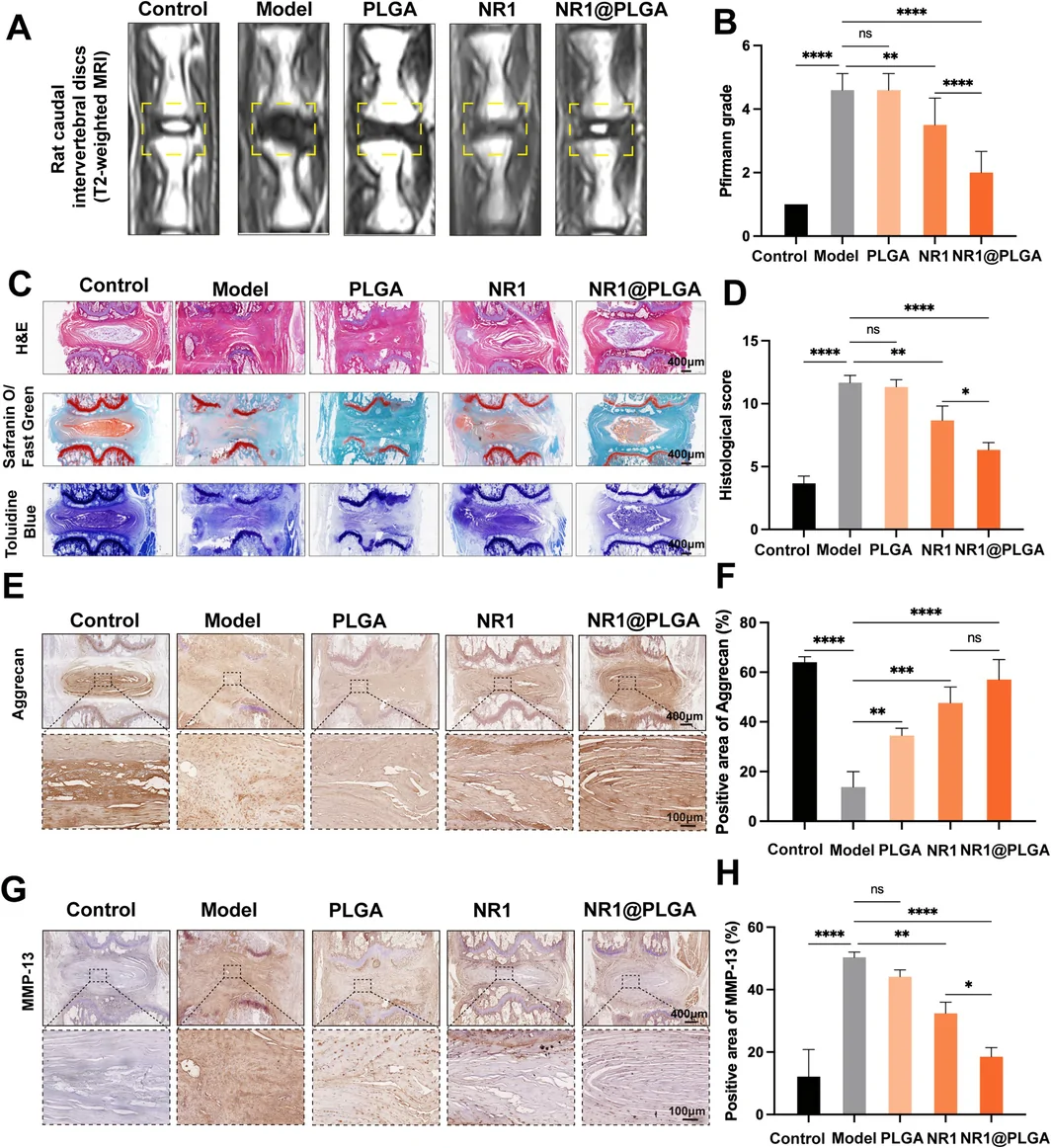
NR1@PLGA protects against IVDD in a rat puncture model. (A) Representative T2‐weighted MRI images of rat caudal intervertebral discs 4 weeks after treatment in the Control, Model, PLGA, NR1 and NR1@PLGA groups. (B) Quantitative analysis of Pfirrmann grades (n=5). (C) Representative H&E, Safranin O/Fast Green and toluidine blue staining of intervertebral disc tissues. (D) Quantitative analysis of histological degeneration scores. (E, F) Representative immunohistochemical staining and quantitative analysis of aggrecan. (G, H) Representative immunohistochemical staining and quantitative analysis of MMP‐13. Data are presented as mean ± SD (*n* = 3). *p < 0.05, **p < 0.01, ***p < 0.001, ****p < 0.0001, ns, not significant.

To assess the therapeutic effectiveness of NR1@PLGA in a living organism, a rat model of IVDD induced by caudal puncture was created and evaluated 4 weeks post‐treatment. Except for the Control group, all groups underwent puncture‐induced modelling. The PLGA, NR1, and NR1@PLGA groups received the corresponding treatments after modelling. T2‐weighted MRI demonstrated that puncture injury caused a pronounced decrease in disc signal intensity and structural deterioration compared with the Control group (Figure 5A). Similar changes were observed in the PLGA group, whereas treatment with free NR1 or NR1@PLGA preserved disc morphology and T2 signal intensity. Pfirrmann grading showed greater protection against disc degeneration in the NR1@PLGA group than in the Model, PLGA, and NR1 groups (Figure 5B). Histological evaluations were performed to further examine structural changes within the intervertebral discs. H&E staining was used to assess overall disc architecture, while Safranin O/Fast Green and toluidine blue staining were applied to evaluate proteoglycan preservation in the extracellular matrix [32, 33]. As shown in Figure 5C, the disc architecture in the Model and PLGA groups was severely disrupted, with a significant loss of the proteoglycan‐rich extracellular matrix. These alterations were alleviated by free NR1 treatment and were further improved in the NR1@PLGA group. Consistent with these observations, quantitative histological scoring showed the lowest degeneration score in the NR1@PLGA group among all puncture‐treated groups (Figure 5D). Because extracellular matrix degradation is closely associated with IVDD progression [34], aggrecan and MMP‐13 expression was analysed by immunohistochemistry. As shown in Figure 5E,F, aggrecan expression was significantly reduced by puncture injury, while treatment with free NR1 or NR1@PLGA significantly increased aggrecan expression (Figure 5E,F). In contrast, the expression of MMP‐13 was elevated in puncture‐induced discs and was more effectively reduced by NR1@PLGA treatment than by free NR1 (Figure 5G,H). These results indicated that using PLGA for delivery improved the therapeutic effectiveness of NR1 in treating IVDD.

### 3.5 In Vivo Safety Assessment of NR1@PLGA

**FIGURE 6:**
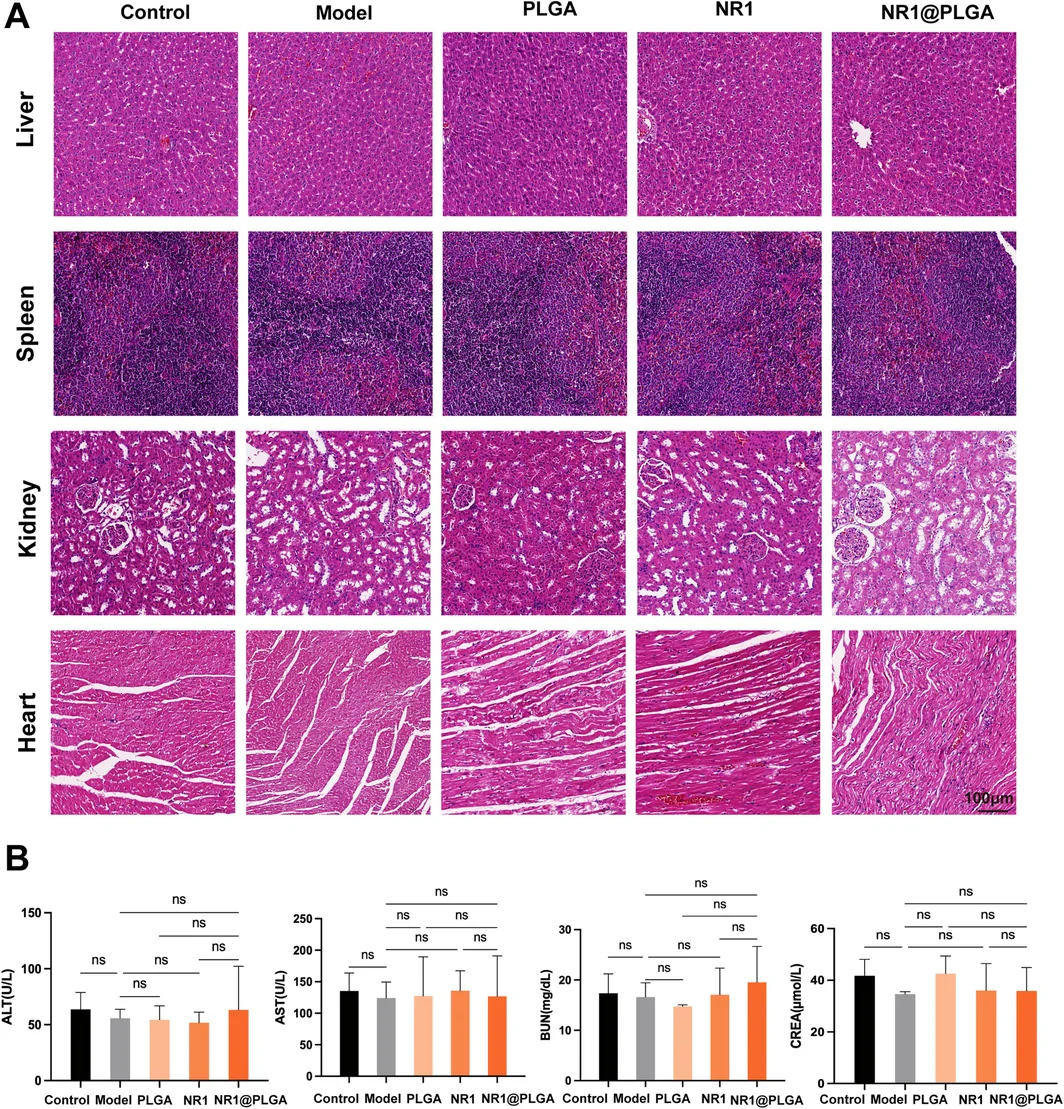
The biosafety evaluation of NR1@PLGA following local administration. (A) Representative H&E staining of the heart, liver, spleen and kidney 4 weeks after treatment. (B) Serum biochemical analysis of liver function markers (AST and ALT) and renal function markers (CREA and BUN). Data are presented as mean ± SD (*n* = 3). ns, not significant.

The in vivo biosafety of NR1@PLGA was examined after administering NR1@PLGA nanoparticles locally into the disc for 4 weeks. H&E staining was used to assess the major organs for any possible tissue damage through histological evaluation. As shown in Figure 6A, no apparent pathological alterations or inflammatory lesions were observed in the heart, liver, spleen, or kidney across all experimental groups. Serum biochemical parameters were further analysed to assess liver and kidney function. Among the five groups, there were no significant differences in the levels of AST and ALT, which represent liver function, or CREA and BUN, which represent kidney function (Figure 6B). These results suggested that local administration of NR1@PLGA was biocompatible and did not cause detectable systemic toxicity in vivo.

## 4 Discussion

Oxidative stress is acknowledged as a significant factor in the progression of IVDD [35, 36]. The accumulation of ROS in excess disrupts the redox homeostasis of cells, causing NPC dysfunction by promoting cellular senescence, apoptosis, and inflammatory responses. These alterations further affect ECM metabolism, characterised by suppressing the production of anabolic matrix components like aggrecan and Col‐2 and promoting the expression of matrix‐degrading enzymes; these pathological changes disrupt ECM metabolism and hasten disc degeneration [37]. In our study, we observed that H_2_O_2_‐induced oxidative stress significantly increased NPC senescence and reduced aggrecan and Col‐2 expression, which was also linked to diminished cell viability. The results further emphasise the key role of oxidative stress in NPC dysfunction and ECM degradation, suggesting that alleviating damage caused by oxidative stress is a viable strategy to delay disc degeneration.

Panax notoginseng has been traditionally utilized to treat bleeding disorders and heart‐related conditions [38]. Its major active components, panax notoginseng saponins (PNS), possess antioxidant, anti‐inflammatory, and cell‐protective properties. Earlier research demonstrated that PNS inhibited oxidative stress‐induced apoptosis of human NPCs and delayed IVDD progression in both cell and animal models [39]. NR1, one of the principal saponins in PNS, has been shown to protect against oxidative stress‐associated cellular injury through its antioxidant, anti‐apoptotic, and anti‐inflammatory activities, together with preservation of mitochondrial function [20, 39]. NR1 has been shown to attenuate H_2_O_2_‐induced oxidative stress by reducing intracellular ROS and mitochondrial oxidative damage, with the involvement of the JNK signalling pathways [20]. In addition, NR1 has been reported to exert neuroprotective effects by reducing oxidative stress [21]. These findings suggested that the antioxidant activity of NR1 may contribute to its protective effects against oxidative stress‐related cellular damage. Recently, NR1 was reported to suppress inflammatory responses and pyroptosis in NPCs by inhibition of the NF‐κB/NLRP3 pathway. It also alleviated IVDD by inhibiting ferroptosis and promoting autophagy, possibly through the STAT3 signalling pathway, thereby preserving disc integrity and delaying disease progression. Consistent with these findings, our results showed that NR1 alleviated H_2_O_2_‐induced NPC senescence and restored extracellular matrix protein expression. Whether these protective effects involve the NF‐κB/NLRP3, STAT3, or other signalling pathways remains unclear and warrants further investigation.

Free NR1 exhibited protective effects against oxidative stress‐induced injury, while PLGA encapsulation further enhanced its biological activity (Figures 4 and 5). PLGA is commonly used as a biodegradable drug delivery carrier with excellent biocompatibility, sustained‐release properties, and it also facilitates intracellular drug delivery [29]. These features are particularly advantageous for the intervertebral disc, where the lack of vascular supply limits drug retention after local administration. In the present study, NR1@PLGA exhibited a sustained‐release profile together with time‐dependent cellular uptake by NPCs (Figures 2E and 3). These properties were associated with reduced cellular senescence, improved preservation of aggrecan and Col‐2 expression, and delayed disc degeneration compared with free NR1. In addition, local intradiscal administration of NR1@PLGA exhibited a favourable safety profile. The major organs did not exhibit any apparent histopathological abnormalities, and the serum biochemical parameters for hepatic and renal function were normal across all groups (Figure 6). These results indicated that administering NR1@PLGA locally was effective therapeutically and did not cause any noticeable systemic toxicity. Our study focused on treatment outcomes over a short period of 4 weeks. As intervertebral disc degeneration is a chronic and progressive disease, more extensive research is needed to verify the lasting effects of NR1@PLGA.

In summary, we demonstrated that PLGA encapsulation increased the treatment efficacy of NR1 for IVDD. Compared to free NR1, NR1@PLGA showed enhanced protection against NPC senescence, better maintained extracellular matrix stability, and more effectively postponed disc degeneration while retaining excellent biocompatibility. The results indicated that administering NR1 locally via PLGA might be an effective strategy for treating IVDD.

## Author Contributions

**Baichuan Zheng:** writing – original draft, methodology, formal analysis, investigation, validation. **Haotian Zhong:** writing – original draft, data curation, formal analysis, validation, methodology. **Zhengwei Wang:** writing – original draft, data curation, formal analysis, methodology. **Junyu Qin:** investigation, validation, methodology. **Ziying Xie:** investigation, validation, methodology. **Rong Tang:** investigation, validation, methodology. **Chengshang Hu:** investigation, validation, methodology. **Xiuming Zhang:** investigation, validation, methodology. **Jianwen Li:** writing – review and editing, conceptualization, project administration, supervision. **Songbo Li:** writing – review and editing, conceptualization, supervision, funding acquisition.

## Funding

This work was supported by Guangdong Basic and Applied Basic Research Foundation, 2021B1515140056. Traditional Chinese Medicine Bureau of Guangdong Province, 20251417.

## Ethics Statement

All animal study was approved by the Experimental Animal Ethics Committee of the Tenth Affiliated Hospital of Southern Medical University (Dongguan People's Hospital) under approval number IACUC‐AWEC‐202605010. The study was conducted in accordance with local legislation and institutional requirements.

## Consent

The authors have nothing to report.

## Conflicts of Interest

The authors declare no conflicts of interest.

## Supporting information


**Figure S1:** Standard curve for HPLC quantification of NR1.
**Figure S2:** CCK‐8 assay to assess the effects of different concentrations of H_2_O_2_ on NPC viability. Data are presented as mean ± SD, ***p < 0.001; ****p < 0.0001, ns, not significant.
**Figure S3:** CCK‐8 assay to assess the cytotoxicity of NR1@PLGA at different concentrations in NPCs.
**Figure S4:** Representative fluorescence images of intracellular ROS detected using DCFH‐DA. Except for the control group, all groups were exposed to H_2_O_2_, and the ‐ NR1, and NR1@PLGA groups received the corresponding treatments .
**Figure S5:** CCK‐8 assay to assess the effects of different concentrations of NR1@PLGA on NPC viability under H_2_O_2_exposure. Data are presented as mean ± SD, ***p < 0.001; ****p < 0.0001, ns, not significant.
**Table S1:** Physicochemical characterization of blank PLGA and NR1@PLGA nanoparticles.

## Data Availability

The data that support the findings of this study are available from the corresponding author upon reasonable request.
