# Supplementary material for: Intradiscal Delivery of Notoginsenoside R1‐Loaded PLGA Nanoparticles Attenuates Intervertebral Disc Degeneration in Rats
Source: J Cell Mol Med. 2026 Sep 23;30(18):e71378. doi: 10.1111/jcmm.71378 (PMC13601857; doi:10.1111/jcmm.71378)
Supplement: Supplementary file 1 [file JCMM-30-e71378-s001.docx]

**Supplementary Information for**

**Intradiscal delivery of notoginsenoside R1-loaded PLGA nanoparticles attenuates intervertebral disc degeneration in rats**

Baichuan Zheng^1,2#^, Haotian Zhong^1#^, Zhengwei Wang^4#^, Junyu Qin^1,5^, Ziying Xie^1^, Rong Tang^1,2^, Chengshang Hu^1,5^, Xiuming Zhang^1,2^, Jianwen Li^1,2,3,5*^, Songbo Li^1,2,3,5*^

^1^The Tenth Affiliated Hospital, Southern Medical University (Dongguan People's Hospital), Dongguan, Guangdong, 523059, China.

^2^Guangdong Medical University, Zhanjiang, Guangdong, 524023, China.

^3^Dongguan Key Laboratory of Basic, Clinical and Digital Research on Common Orthopedic Diseases, Dongguan, Guangdong, 523059, China.

^4^The Affiliated Dongguan Songshan Lake Central Hospital, Guangdong Medical University, Dongguan GuangDong 523326, China.

^5^Shenzhen School of Clinical Medicine, Southern Medical University, Shenzhen, 518000, China.

Correspondence: Jianwen Li (lijianwen806@smu.edu.cn), Songbo Li (imsongbo@smu.edu.cn)

\# These authors contributed equally to this work.

**Supplementary Figure 1.** Standard curve for HPLC quantification of NR1.

**Supplementary Figure 2.** CCK-8 assay to assess the effects of different concentrations of H₂O₂ on NPC viability. Data are presented as mean ± SD, ****p* < 0.001; *****p* < 0.0001, *ns*, not significant.

**Supplementary Figure 3.** CCK-8 assay to assess the cytotoxicity of NR1@PLGA at different concentrations in NPCs.

**Supplementary Figure 4.** Representative fluorescence images of intracellular ROS detected using DCFH-DA. Except for the control group, all groups were exposed to H₂O₂, and the PLGA, NR1, and NR1@PLGA groups received the corresponding treatments under H₂O₂ stimulation.

**Supplementary Figure 5.** CCK-8 assay to assess the effects of different concentrations of NR1@PLGA on NPC viability under H₂O₂exposure. Data are presented as mean ± SD, ****p* < 0.001; *****p* < 0.0001, *ns*, not significant.

| **Formulation** | **Hydrodynamic size (nm)** | **PDI** | **Zeta potential (mV)** | **Drug loading** | **Encapsulation efficiency** |
| --- | --- | --- | --- | --- | --- |
| PLGA | 197.82±17.90 | 0.23±0.015 | −18.96±0.60 | N/A | N/A |
| NR1@PLGA | 198.20±17.93 | 0.22±0.02 | −24.79±3.18 | 5.60 | 61.04 |

**Table S1.** Physicochemical characterization of blank PLGA and NR1@PLGA nanoparticles.
